# Severe Vitamin D Deficiency—A Possible Cause of Resistance to Treatment in Psychiatric Pathology

**DOI:** 10.3390/medicina59122056

**Published:** 2023-11-21

**Authors:** Adela Magdalena Ciobanu, Cristian Petrescu, Cristina Anghele, Mihnea Costin Manea, Constantin Alexandru Ciobanu, Diana Mihaela Petrescu, Mihalache Oana Antonia, Sorin Riga

**Affiliations:** 1 Neuroscience Department, Discipline of Psychiatry, Faculty of Medicine, “Carol Davila” University of Medicine and Pharmacy, 020021 Bucharest, Romania; adela.ciobanu@umfcd.ro (A.M.C.); cristian.petrescu@drd.umfcd.ro (C.P.); romcrys@yahoo.com (C.A.); mihnea.manea@live.com (M.C.M.); 2 Department of Psychiatry, “Prof. Dr. Alexandru Obregia” Clinical Hospital of Psychiatry, 041914 Bucharest, Romania; 3 Faculty of Medicine, University of Medicine and Pharmacy “Carol Davila”, 020022 Bucharest, Romania; 4 Department of Neurology, “Carol Davila” University of Medicine and Pharmacy, 020021 Bucharest, Romania; diana-mihaela.vlad@rez.umfcd.ro; 5 Neurology Clinic, “Fundeni” Clinical Institute, 022328 Bucharest, Romania; 6 Department of Stress Research and Prophylaxis, “Prof. Dr. Alexandru Obregia” Clinical Hospital of Psychiatry, 041914 Bucharest, Romania; d_s_riga@yahoo.com; 7 Romanian Academy of Medical Sciences, 927180 Bucharest, Romania

**Keywords:** vitamin D deficiency, mental health, depression, depressive symptoms, anxiety, mood

## Abstract

In the last few years, vitamin D functions have been studied progressively, and along with their main role in regulating calcium homeostasis, the potential function in the nervous system and the link between different psychiatric disorders and vitamin D deficiency have been revealed. The discovery of vitamin D receptors in multiple brain structures, like the hippocampus, led to the hypothesis that vitamin D deficiency could be responsible for treatment resistance in psychiatric diseases. The aim of this study was to analyze the current knowledge in the literature regarding vitamin D deficiency among individuals afflicted with psychiatric disorders and assess the potential therapeutic benefits of vitamin D supplementation. A systematic search was conducted on the PubMed database for articles published in the last five years (2016–2022) in English, focusing on human subjects. Results show that vitamin D deficiency has implications for numerous psychiatric disorders, affecting mood and behavior through its influence on neurotransmitter release, neurotrophic factors, and neuroprotection. It also plays a role in modulating inflammation, which is often elevated in psychiatric disorders. In conclusion, vitamin D deficiency is prevalent and has far-reaching implications for mental health. This review underscores the importance of exploring the therapeutic potential of vitamin D supplementation in individuals with psychiatric disorders and highlights the need for further research in this complex field.

## 1. Introduction

Since the discovery of vitamin D in 1921 and its active form, 1,25-dihydroxyvitamin D (1,25-(OH)_2_D) [1], in 1967, its role in bone health and calcium homeostasis has been well established. However, recent biochemical advances have increased interest in its non-skeletal function, given the prevalence of its deficit in more than 50% of commonly encountered patients in clinical practice [2].

Vitamin D can be found in two forms: ergocalciferol (Vitamin D2), created in plants such as mushrooms but also available in supplements; and cholecalciferol (Vitamin D3—the “sunshine” vitamin), obtained from skin sunlight radiation, exposure to ultraviolet B, oral supplements, and various foods (juices, milk, cereals, oily fish). After intestinal absorption, they are metabolized in the liver and kidneys, resulting in calcidiol (25-hydroxycholecalciferol), the body’s storage of vitamin D, and calcitriol (1,25-dihydroxyvitamin D), also known as the active metabolite. Thus, calcidiol is used for the measurement of total body vitamin D, with a normal range between 25 ng/mL and 80 ng/mL [3].

**Figure 1:**
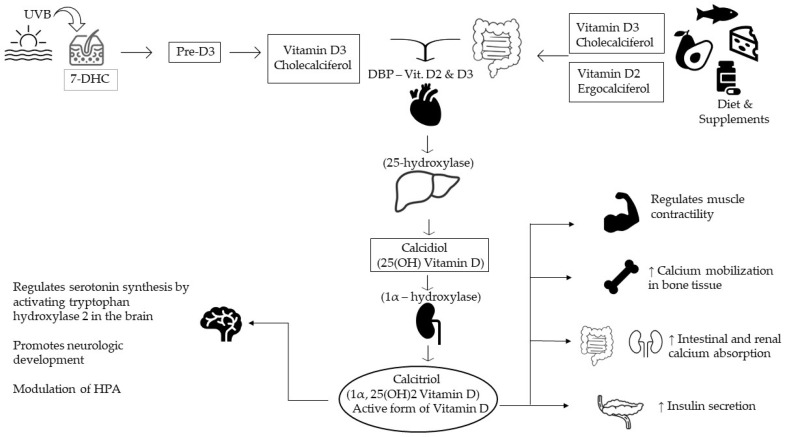
Vitamin D synthesis and its main functions. 7-DHC—7-dehydrocholesterol; DBP—vitamin D binding protein; HPA—hypothalamic-pituitary-adrenal axis.

Vitamin D plays a pivotal role in regulating calcium and phosphorus levels within the body. Its functions are multifaceted, including the stimulation of calcium and phosphorus absorption in the intestines, mobilization of calcium from bones, and increased reabsorption of calcium in the distal tubule of the kidneys (Figure 1). Notably, these actions on bone and, to some extent, the kidneys are modulated by the parathyroid hormone [4]. One crucial aspect of the vitamin D endocrine system is its ability to establish a robust feedback mechanism. In this mechanism, 1,25(OH)2D3, an active form of vitamin D, acts to inhibit the expression of the CYP27B1 gene in the kidney. This, in turn, leads to a decrease in the production and secretion of parathyroid hormone (PTH) by the parathyroid gland and an increase in the expression of fibroblast growth factor 23 (FGF23) in the bone [5]. This intricate feedback loop is vital for maintaining the delicate balance of calcium and phosphorus in the body.

In recent discoveries, vitamin D receptors have been found in the brain (hippocampus, substantia nigra, and cerebellum), which led to further explanations for their role in neurologic development, psychiatric conditions (anxiety, depression, psychoses), or behavior. An additional role could be the involvement in modulation of the hypothalamic-pituitary-adrenal axis (HPA), thus regulating the production of epinephrine, norepinephrine, and dopamine [6,7]. Thirdly, this hormone has neuroprotective effects through multiple mechanisms, such as the reduction of nitric oxide synthesis and calcium toxicity, protection of the neurons from induced cell death, antioxidant properties, and modulation of cytokine release [8]. The discovery of vitamin D receptors in the brain has prompted research into its potential role as a neuroactive steroid in regulating neurophysiological processes associated with depression [9]. Low levels of circulating vitamin D (25-hydroxyvitamin D3; 25(OH)D) have been linked to depression [10]. In the brain, vitamin D is biologically active (1,25-dihydroxyvitamin D3), and it interacts with the nuclear vitamin D receptor (VDR) and enzymes involved in its activation and metabolism. This suggests that vitamin D may have autocrine or paracrine effects on the brain [8]. It may counteract the hyperactivity of the hypothalamic-pituitary-adrenal (HPA) axis and cortisol overproduction, common in depression due to reduced glucocorticoid receptor sensitivity in the brain [11].

It has also been found that neonatal deficiencies of vitamin D in rats alter the shape and size of the brain, growth factor expression, cell proliferation, enlargement of the ventricles, and cortical thinning [7,8,9,10,11,12].

The pervasive issue of vitamin D insufficiency and deficiency is a global concern, affecting approximately half of the world’s population, irrespective of age or ethnicity, as highlighted in a seminal study by Nair and Maseeh [13]. According to a large-scale study involving 14,302 adults aged from 18 to 65 years, the prevalence of vitamin D insufficiency and deficiency was 32.7% and 41.9%, respectively [14]. Notably, this nutritional challenge has significant implications for public health, as underscored by a prominent meta-analysis conducted by Garland et al. (2014) [15]. Their extensive analysis revealed a compelling association between low serum levels of 25-hydroxyvitamin D (25(OH)D) and an elevated risk of all-cause mortality. This finding underscores the far-reaching impact of vitamin D status on overall health and underscores the importance of addressing and mitigating this prevalent deficiency to enhance global well-being.

The intricate relationship between vitamin D and mental health disorders remains a topic of ongoing investigation, despite the existing body of biochemical knowledge. Several factors contribute to this complexity. Firstly, individuals who are hospitalized or experiencing social withdrawal as a consequence of psychiatric illnesses may exhibit low levels of vitamin D [16]. This deficiency can be attributed to reduced sun exposure as well as dietary restrictions commonly associated with these conditions. Secondly, vitamin D is a fat-soluble compound, and in individuals with obesity, its circulatory levels may be diminished [17]. This decrease in circulating vitamin D can be attributed to its redistribution within the body’s fat stores, which hinders its availability for physiological functions. Furthermore, the use of atypical antipsychotic medications, often prescribed to individuals with mental health disorders, can lead to weight gain, exacerbating the challenge of maintaining adequate vitamin D levels. As such, the interplay between vitamin D status and mental health represents a multifaceted area that necessitates further exploration to elucidate its intricacies and potential therapeutic implications [18].

In light of the multifaceted nature of the subject matter, the primary objective of this study is to conduct a thorough and critical review of the existing body of literature concerning vitamin D deficiency among individuals afflicted with psychiatric disorders. A central focus lies in evaluating the potential advantages and therapeutic efficacy of prophylactic vitamin D interventions as a means of ameliorating mental health outcomes among affected populations. Our secondary objective is to evaluate the role of vitamin D in the pathogenesis of mental disorders and its therapeutic implications. Furthermore, this study is committed to scrutinizing the most recent publications and cutting-edge research findings in this domain, ensuring that the synthesis of knowledge is up-to-date and reflective of the dynamic landscape of vitamin D-related psychiatric research.

## 2. Materials and Methods

For the purpose of this scoping review, we conducted an analysis using the PubMed database in April 2022, searching for vitamin D cross-referenced with the Diagnostic and Statistical Manual (DSM) 5th edition [19] standard classification of mental disorders. We included full articles published in the last 5 years (2016–2022) in order to comprise the most recent data, written in English, with human subjects.

The list of chapters that we included were: neurodevelopmental diseases, psychotic spectrum diseases (e.g., schizophrenia), bipolar disorder, depressive disorders, anxiety disorders, obsessive-compulsive disorders, trauma and stress-related disorders, dissociative disorders, somatic disorders, eating disorders, elimination disorders, sleep-wake disorders, sexual disorders, gender dysphoria, disruptive behavior and impulse control diseases, substance abuse disorders, neurocognitive diseases, personality disorders, paraphilia, and applied vitamin D supplementation.

We excluded articles based on the following criteria: Studies conducted in animal models and studies assessing the influence of combined multiple nutrients supplementation.

The studies that were identified underwent a thorough verification process to eliminate duplicates. Subsequently, two researchers independently evaluated these studies based on their titles to determine their eligibility. Following this initial assessment, the next step involved an independent evaluation of the studies’ abstracts by the same researchers to further assess their eligibility. In case of any discrepancies or disagreements at any stage, a discussion between the two researchers was held to resolve them. Finally, the full texts of the studies that met the eligibility criteria were extracted for further analysis.

## 3. Results

### 3.1. Description of the Included Articles

Our search yielded a substantial number of publications, comprising more than 100 articles, all of which explored the influence of vitamin D on mental health. This investigation uncovered implications for nearly 70% of the psychiatric pathologies listed in the DSM-5, a testament to the potential significance of vitamin D in the realm of mental well-being. Among the 19 distinct disorders encompassed by our search, seven exhibited no discernible outcomes when cross-referenced with vitamin D. These disorders included somatic diseases, disruptive behavior and impulse control disorders, dissociative disorders, gender dysphoria, substance abuse disorders, paraphilic disorders, and personality disorders. The outcomes for the remaining 12 disorders are explained in the subsequent sections.

### 3.2. Psychiatric Diseases and Vitamin D

Vitamin D exhibits a remarkable ability to traverse the blood-brain barrier, activate vitamin D receptors within the central nervous system, and exert influence over human behavior regulation, as elucidated by studies such as Farhangi et al. (2017) [20]. Its role extends to the intricate modulation of neurotransmitter release and the synthesis of neurotrophic factors, pivotal mechanisms contributing to the improvement of mood and behavioral outcomes in individuals, as highlighted in the research conducted by Macova et al. (2017) [21]. This multifaceted influence can also be attributed to vitamin D’s neuroprotective properties, with evidence indicating its potential to reduce plasma C-reactive protein levels in psychiatric disorder patients and its capacity to modulate inflammation through the suppression of proinflammatory cytokines, as observed in studies like Jamilian et al. (2019) and Barker et al. (2013) [22]. These findings collectively underscore the pivotal role of vitamin D in neurological and psychiatric well-being, shedding light on its potential therapeutic implications.

After crossing the blood-brain barrier, vitamin D has implications for many processes: cell differentiation, neurotransmitter synthesis, neurotrophic production and release, calcium homeostasis, cognitive function, oxidative damage prevention, and neuronal structure-function. Therefore, it is not surprising that low vitamin levels have been associated with various psychiatric disorders (depression, schizophrenia, autism spectrum disorders, and Alzheimer’s disease [23].

Some data suggest that the psychiatric population has a higher rate of vitamin D deficiency than the general population [17,23,24,25,26,27,28,29,30,31]. Patients from a psychiatric ward in Serbia have been included in a cross-sectional design study, and a significant number of patients had a lower vitamin D level than expected [30]. Even in an equatorial nation, despite the geographic location and outdoor activities, levels of vitamin D were still low among psychiatric hospitalized subjects [29].

All things considered, in 2017, Goluza I. et al. tried answering a simple question as to whether or not vitamin D levels should be utilized in the screening methods for chronic mental illness. They observed the results of an audit with 300 psychiatric patients, of whom only 12% had vitamin D tested; therefore, they reported screening inadequacy since there is a high rate of deficit in general psychiatric inpatients [28]. Similarly, the findings of another study show gaps in the evaluation of vitamin D deficiency and inadequate treatment supplementation [27].

Iknonen H. et al. used the Finland birth cohort from 1966, and they explored the results of almost 5000 subjects with vitamin D and psychiatric illnesses (depression, schizophrenia, and other psychoses) and found a negative correlation in schizophrenia patients between vitamin D and smoking [26].

Vitamin D supplementation therapy in psychiatric patients had beneficial effects on depressive symptoms, biomarkers of inflammation, and even oxidative stress [32,33,34] C-reactive protein and total antioxidant levels have been improved [34].

Patients with alexithymia have been included in a study for measuring serum levels of vitamin D and genotyping vitamin D binding protein, and their results suggest that lower vitamin levels could be involved in the pathophysiology of the disease [35].

### 3.3. Neurodevelopmental Disorders

The majority of people with intellectual disabilities were reported to have deficient or insufficient vitamin D levels, with a higher prevalence in women [36]. The level of vitamin D deficiency in patients with intellectual disabilities was not correlated with seasonality, type of admission, or associated treatment [37]. Deficient levels of maternal vitamin D were not correlated with a higher risk of intellectual disability or autism spectrum disorders [38].

Deficient levels of vitamin D in newborns were not correlated with a higher risk of autism spectrum disorders [39]. Autistic children had a significantly lower level of vitamin D when compared to healthy children [40]. Vitamin D had a positive effect on irritability symptoms in autistic children [41].

Vitamin D levels were significantly lower in ADHD children and adolescents [42]. Vitamin D supplementation could improve mental health and behavioral symptoms in children with ADHD and low vitamin D levels [43]. Another study found that vitamin D supplementation in ADHD patients improved outcomes such as cognitive function, hyperactivity, and inattention.

### 3.4. Psychotic Spectrum Diseases

Epidemiologic evidence suggests that the etiology of schizophrenia includes genetic and environmental factors and common risk factors for vitamin D, including low prenatal vitamin D, children born in winter and early spring, high-altitude residents or urban residents, and dark-skinned migrants who move to cold climates [44]. Also, schizophrenia patients have poorer general health; they are less active, have a poor diet, and have an increased risk of medical conditions [45].

Studies show a strong association between schizophrenic patients and vitamin D deficiency [23,44,45,46,47,48]. One study with over 300 participants aimed to investigate psychotic disorders and found lower vitamin D levels in this group of subjects, speculating that these findings could be a consequence of the negative symptoms of the disease, explained through withdrawal, inactivity, and, therefore, less exposure to sunlight [49].

A UK cross-sectional study with 324 English subjects identified that over 90% of people with psychosis were diagnosed with suboptimal vitamin D deficiency, schizophrenia patients had rates of 50%, and comparable rates were found in first-episode psychosis [46].

Salavert J. et al. studied first-episode psychosis, determining vitamin blood levels in a group of 45 subjects, after 6 months of follow-up, they were divided into two subgroups (schizophrenia versus psychosis), and the results showed that the vitamin D deficit was lower in the schizophrenia group [47].

Recent studies have initiated vitamin D supplementation therapy as an augmented treatment to the standard regimen for schizophrenia [50,51,52]. Some of them found a negative, but not significant, association between vitamin D and different subscale scores (Positive and negative syndrome scale—PANSS), and other studies have reported improved cognition but not psychotic symptoms [50,51]. Later on, in 2021, the data obtained suggested that both antipsychotic medication and vitamin D treatment may improve positive and negative symptoms and also improve attention span [51].

### 3.5. Bipolar Disorders

Recent studies have shown that there might be a link between bipolar disorder and vitamin D deficiency [53]. There is little information concerning vitamin D levels and supplementation effects on the course of bipolar disorder; however, there has been a noted increase in 25OHD synthesis during BD decompensation [54]. Other studies have shown a lower level of vitamin D in people with bipolar disorders, but supplementation was not correlated with an improvement in depressive symptoms [55].

### 3.6. Depression

Antidepressants, as indicated by Cipriani et al. in 2018 [56], can indeed serve as a viable remedy for depression. Nevertheless, their effectiveness in addressing the condition may fall short in certain cases. Additionally, it’s not uncommon for individuals to experience a recurrence of symptoms during treatment, often necessitating multiple trials with different antidepressants before achieving a satisfactory response, as noted by Rush et al. in 2006 [57]. Therefore, it is imperative to explore supplementary treatment alternatives for individuals grappling with depression.

While the pathophysiology of depression is not yet fully understood, a deficit in vitamin D serum levels is linked to a higher likelihood of having depression, even after adjusting for confounding variables such as time of the year, socioeconomic status, and lifestyle [58]. Depression is also associated with low-grade inflammation, elevated cytokine levels, and stress-induced disruptions in lipid and glucose metabolism. Vitamin D has been proposed to act as a glucocorticoid antagonist, potentially protecting the hippocampus during HPA axis dysregulation [59]. Additionally, it may influence hippocampal neuron development, serotonin and dopamine release, and synaptic plasticity mechanisms through various pathways [60,61]. Furthermore, circulating 25(OH)D levels have been linked to the body’s immune responses [62].

The serum levels of vitamin D were correlated with the degree of depression as measured by scores such as the Hospital Anxiety and Depression Scale, with a stronger correlation in male patients as opposed to female patients [63,64]. While the correlation between vitamin D levels and depression has been studied more and more, depressive symptoms do not appear in all patients suffering from vitamin D deficiency, and not all patients with vitamin D deficiency suffer from depression, indicating a more complex pathophysiological pathway.

Vitamin D supplementation was correlated with a decrease in depressive symptoms in a variety of populations, such as elderly depressed people [65], patients with metastatic pulmonary cancer and comorbid depression [66], adolescent girls suffering from depression, and patients with acute stroke and comorbid depression [67]. Vitamin D supplementation was not correlated with a significant difference in neurotransmitters such as serotonin or oxytocin, indicating a different pathophysiological mechanism [65]. Several clinical trials have shown vitamin D supplementation to be an effective way of treating depression [68], even though when antidepressant treatment and psychotherapy are applied in an adequate scenario, the effect of vitamin D supplementation can be negligible [69,70] or at most moderate [71].

The outcomes of a comprehensive meta-analysis present compelling evidence pointing to a significant association between low circulating vitamin D levels and a notable three-fold increase in the susceptibility to post-stroke depression. Furthermore, the study underscores several other risk factors, including female gender, hyperlipidemia, and higher NIHSS scores, which were also identified as contributors to an augmented risk or occurrence of post-stroke depression. These findings illuminate the importance of considering routine screening for circulating vitamin D concentrations, not only in individuals recovering from stroke but also in other high-risk populations. Such proactive measures may aid in early identification and intervention, potentially improving mental health outcomes in these vulnerable groups, as documented in the study by Hung et al. (2023) [72].

### 3.7. Anxiety Disorder

Since epidemiological research has evidenced a relationship between vitamin D and depression, and given the usual association of depressive symptoms in patients suffering from anxiety, studies have aimed to research the therapeutic effect of vitamin D supplementation in such patients [44,73,74,75].

This current evidence shows several potential mechanisms for improvement after vitamin D supplementation, such as the role in calcium homeostasis or the interference in serotonin synthesis by expression of the serotonin synthesizing gene, therefore maintaining normal levels. Another function is the mediation of several pathways for insulin or serotonin, which are associated with mood disorders [76].

In an experiment conducted by Zhu, C. et al., approximately 160 Chinese individuals suffering from depression were subjected to supplementation with vitamin D for 6 months. There was no perceivable impact on depressive symptoms in these patients, but an improvement in anxiety symptoms [44].

In 2019, Eid, A. et al. investigated the effects of vitamin D supplementation on generalized anxiety disorder (GAD), clinically and biologically, on 30 diagnosed patients from Saudi Arabia. The individuals had serum vitamin D, serotonin, and neopterin (a mediator for cellular immunity and a biomarker of oxidative stress) measured and were divided into 2 groups (with or without vitamin D supplementation). The researchers found significant improvement in GAD patient scores in the vitamin D-treated group [73].

On the other hand, Casseb G.A.S. et al. and De Koning E.J. et al. have described ambiguous results on this matter with little association between vitamin D therapy and anxiety, independently from depression. The results showed an association between anxiety symptoms and serum vitamin D or depressive symptoms. However, the relationship between vitamin D levels and anxiety symptoms was explained by demographic and lifestyle factors such as sunlight, food, or supplements [74,75].

### 3.8. Obsessive-Compulsive Disorder

Recent investigations have shown an association between reduced vitamin D levels and obsessive-compulsive disorder (OCD) [77,78]. It is noteworthy that certain studies, despite not discerning significant differences in vitamin D levels between individuals diagnosed with OCD and healthy controls, have reported that even the healthy participants displayed vitamin D levels within the range considered a deficiency [79]. Although a pathophysiological connection between vitamin D deficiency and OCD has been posited, the precise mechanisms underlying this relationship remain elusive [80].

### 3.9. Trauma and Stress-Related Disorders

While the incidence rate for traumatic events during one’s lifetime varies between 25% and 80%, only a small group of trauma victims develop post-traumatic stress disorder (PTSD). This disease is associated with cardiovascular complications, depression, and anxiety [81]. Vitamin D may be implicated through three different mechanisms in this pathogenesis: Firstly, its neuro-inflammatory and neuro-immunological regulation may be involved with psychiatric disorders from PTSD [82]. Secondly, brain regions with altered activity in patients with PTSD express vitamin D receptors (prefrontal cortex, cingulate cortex, hypothalamus) [9]. Thirdly, vitamin D is implicated in the regulation of serotonin and catecholamine [83]. In a study by Terock J. et al., the researchers analyzed a population-based prospective cohort in Germany. Multivariable logistic regression has revealed that vitamin D levels are associated inversely with PTSD, suggesting that vitamin D deficiency and functional polymorphisms of vitamin D binding protein were related to increased odds of PTSD (OR = 2.02; *p* = 0.028) [35].

Another study investigated vitamin D supplementation during winter and the effect on biological markers of stress resilience (serotonin, cortisol, psychophysiological activity) in a randomized clinical trial. In this group, resilience to stress varied with seasonal changes in vitamin D levels (vitamin supplementation during the winter influenced resilience to stress in the spring) [84].

### 3.10. Eating Disorders

Patients suffering from eating disorders, such as anorexia nervosa (AD), have been found to present deficient levels of vitamin D [85], with some studies indicating that over half the patients have vitamin D deficiency [86]. While vitamin D deficiency is more of a result than a cause of AD, it has been suggested as a potential cause for the depressive symptoms seen in such patients [87], though this effect has been contested [88]. Vitamin D supplementation for eating disorders should be considered more on the grounds of the comorbid pathologies, such as osteoporosis, than for the eating disorder itself [89].

### 3.11. Elimination Disorders

The evaluation of urinary incontinence has led to an association between vitamin D and conditions that increase the risk for elimination disorders. Vitamin D receptors can be found in the bladder and pelvic floor muscles. Additionally, prostatic cells can express a hydroxylase that can synthesize the active form of vitamin D [90].

A recent study evaluated the relationship between vitamin D and urinary incontinence in a longitudinal observation of a cohort of older adults with a previous assessment of daily activities and cognitive and depression screening tests and found that more than half of the participants were deficient in vitamin D, and among them, 38% developed urinary incontinence. This study demonstrates the association between vitamin D and optimal pelvic floor function [91].

### 3.12. Sleep-Wake Disorders

Recent studies have shown that vitamin D levels play a role in regulating sleep patterns and, consequently, in sleep disorders [92]. Higher vitamin D levels were significantly associated with a shorter time required to fall asleep [93]. It is worth mentioning that differences in sleep patterns and duration related to vitamin D levels were only observed in daytime workers and not in nighttime workers [94]. Vitamin D deficiency has been associated with lower sleep duration and worse sleep quality in both adult and pediatric populations, with a subsequent increase in the time of sleep onset in pediatric populations, suggesting an effect of vitamin D on circadian rhythm regulation [95].

There appears to be an inverse relationship between excessive daytime sleepiness and vitamin D levels [96]. Lower levels of vitamin D have been reported in people suffering from narcolepsy with cataplexy [97]. Patients with chronic insomnia had significantly lower levels of vitamin D when compared to healthy controls [98]. Patients who were not responding to classical treatment had even lower levels of serum vitamin D when compared with treatment responders [98].

Vitamin D deficiency was present in a large proportion of patients with obstructive sleep apnea (OSA) [99]. Reduced vitamin D levels were found in pediatric patients with obstructive sleep apnea, with lower levels also observed in African American children and obese children, these factors having a cumulative effect [100]. Subjects with OSA have a higher risk of vitamin D deficiency, which is in turn independently correlated with insulin resistance in these patients [101]. Vitamin D levels are inversely correlated with OSA severity [102]. Continuous positive airway pressure (CPAP) treatment improved the clinical manifestations of OSA and vitamin D levels in obese patients [103].

### 3.13. Sexual Disorders

Among the organic causes of erectile dysfunction, vascular diseases are the most common (predominantly atherosclerosis). Low levels of vitamin D are associated with increased risk for atherosclerotic events through inflammation, endothelial dysfunction, atherosclerosis, and impaired glucose homeostasis [103].

Apart from the well-known calcium effects, several studies have conducted further analysis of the role of vitamin D levels and sexual function [104,105,106,107,108].

In a randomized, double-blind study with female participants diagnosed with sexual dysfunction and serum vitamin D deficiency, after intramuscular administration of vitamin D and evaluation of depressive symptoms, the results showed an improvement in sexual function. Furthermore, the effect of this treatment was independent of the effect on depression [105].

In comparison, another study aimed to investigate the effect of lower vitamin D levels and sexual function in men and concluded that, compared to the healthy participants, the subjects with low vitamin D obtained lower scores in erectile function, sexual desire, and orgasmic function, suggesting that low vitamin D impairs male sexual functioning, and also concluded that the degree of hypovitaminosis correlates with the severity of sexual dysfunction [106]. Canguven O. et al. conducted research with 102 male participants with vitamin D deficiency who received treatment for 12 months while being monitored. The results suggested that the treatment improved testosterone function, erectile function, and metabolic syndrome in middle-aged men [104]. Another large representative sample of U.S. men was included in a cross-sectional study where vitamin D deficiency has been associated with erectile dysfunction but is independent of atherosclerosis [107].

### 3.14. Neurocognitive Diseases

In the last 25 years, vitamin D has become a candidate for the development and function of the nervous system and a therapeutic tool [108]. Vitamin D receptors are found in the hippocampus and, therefore, play an important role in memory formation. Patients with vitamin D deficiency present with small hippocampal volumes. The active form of vitamin D supports neurotransmission, neuroprotection, and synaptic plasticity [109].

Studies have shown that patients with Alzheimer’s disease have lower concentrations of vitamin D [41,110,111,112,113,114,115]. Moreover, low serum levels of vitamin D are associated with a higher degree of cognitive impairment [116]. A 1 nmol/L decrease in serum vitamin D was associated with a 6% risk of Alzheimer’s disease [117].

Digging deeper and comparing visual with verbal memory, Kuzma E. et al. studied severe vitamin D deficiency in dementia patients and found an association with only visual memory decline [118]. In a case-control study with geriatric patients, serum vitamin D levels were tested, and a lower concentration was linked to delirium [119].

On the other hand, vitamin D has shown no protective effect on cognitive function in a 5-year follow-up study. Over the course of 5 years, 661 dementia-free patients were included in the study, and only 141 subjects developed dementia. The authors found that, among women, a 50% increase in vitamin D concentration was associated with a higher risk of dementia. In comparison, in a study with 180 patients with already-diagnosed mild cognitive impairment, a 12-month vitamin D supplementation improved cognition by reducing oxidative stress [117]. Another study showed that black participants benefited from vitamin supplementation and showed cognitive improvement in executive and attention scores [120].

## 4. Discussion

The relationship between vitamin D supplementation and mental health is complex and challenging. While a significant number of high-quality studies have suggested a positive impact of vitamin D supplementation on depression, the same level of support is not universal for all mental health problems. The study conducted by Guzek et al. in 2021 has shed light on this intricate relationship, emphasizing that vitamin D supplementation should not be viewed as a singular dietary intervention for the prevention and treatment of mental health disorders. Instead, a comprehensive approach is warranted, encompassing broader dietary modifications to ensure adequate vitamin D intake through natural food sources [121]. This approach aligns with previous systematic reviews that underscore the importance of diet in mental health, such as the effects of fruit and vegetable consumption and various dietary patterns. Additionally, promoting physical activity, a confirmed factor associated with mental health further complements the potential positive influence of vitamin D [122]. However, it is essential to consider that some studies lack detailed information on concurrent interventions and factors like calcium supplementation and antidepressant use, which may impact the outcomes. Therefore, the multifaceted nature of this relationship necessitates continued research to refine our understanding and develop effective strategies for managing mental health.

Thus, it is unclear whether vitamin D deficiency in the psychiatric population is a consequence of mental illness or if it has a role in the pathogenesis. Since sunlight is an essential element for vitamin D synthesis, it is expected that inpatients with institutionalization, social isolation, or multidrug regimen will have lower levels of vitamin D [24].

A comprehensive meta-analysis conducted by Xie et al. in 2022 [123] provides valuable insights into this matter. The findings consistently highlight the relevance of serum 25(OH)D levels, dosage of vitamin D supplementation, body mass index (BMI), gender, and intervention duration.

One of the key takeaways from this analysis is the significance of serum 25(OH)D levels. Whether individuals are currently experiencing depression or not, those with low vitamin D levels stand to benefit the most from vitamin D supplementation. This suggests that vitamin D may play a crucial role in addressing mental health concerns, especially for those with pre-existing deficiencies.

Furthermore, gender appears to be a determining factor in the effectiveness of vitamin D supplementation. Females, in particular, exhibit a higher likelihood of experiencing positive outcomes from such interventions. This insight underscores the importance of considering gender-specific approaches when addressing mental health issues.

Regarding dosage and intervention duration, the meta-analysis suggests that a daily supplementary dose of 2800 IU and an intervention period of 8 weeks represent key thresholds for observing the beneficial effects of vitamin D. These findings provide valuable guidance for healthcare professionals in designing effective vitamin D supplementation strategies.

Additionally, the research reveals distinctions in how vitamin D supplementation impacts different weight groups. Normal-weight individuals appear to benefit from both the prevention and treatment of depression through vitamin D supplementation. However, for those who are overweight, the benefit primarily lies in the treatment rather than the prevention of depression.

Several mechanisms are implicated in the beneficial effect of vitamin D on brain function and, therefore, in psychiatric diseases. These are illustrated in Table 1.

To our knowledge, this is the first review that compared all psychiatric diseases and analyzed the known information on vitamin D deficiency’s influence. Psychotic spectrum disease, depression, and neurodevelopmental and neurocognitive diseases were the ones with the highest rate of implications. Our results are similar to those of Itzhaky D. et al. and Jamilian H. et al., where schizophrenia patients were compared with depression patients and healthy control subjects, with the former having lower levels of vitamin D [130,131].

Taking into consideration the effect of vitamin D deficiency on the body, there is a possibility that supplementation treatment will be beneficial for psychiatric patients, but at this moment, there is a necessity for longitudinal studies on this matter. Firstly, current literature suggests there is a gap in vitamin detection for psychiatric patients, and secondly, these types of patients are not the most compliant with treatment; therefore, additional medication might not be successful in the long term. Surely, by supplementing vitamin D in deficient patients alongside antipsychotic treatment, we should see some clinical improvement [27,28].

The systematic review and meta-analysis conducted by Tuomas Mikola et al. in 2022 offer valuable insights into the potential therapeutic effects of vitamin D supplementation on depressive symptoms in adults. While the findings are promising, several important considerations emerge from this analysis that warrant careful attention [132].

Firstly, the study challenges the traditional cutoff level used to define vitamin D deficiency, suggesting that for addressing neuropsychiatric disorders, higher serum 25(OH)D concentrations may be necessary. The standard cutoff of 50 nmol/L may not suffice to unlock the full neuroprotective potential of vitamin D, as indicated by research in conditions such as multiple sclerosis [132].

Another crucial aspect is determining the optimal therapeutic window for vitamin D in relieving depressive symptoms. The study highlights the need to strike a balance, as extremely high doses of vitamin D could potentially limit its benefits. This limitation may occur through immunomodulatory effects that lead to secondary hypercalcemia in the central nervous system [132].

The duration of vitamin D supplementation is also a factor influencing its effectiveness. While eight weeks may be considered sufficient for a response, shorter interventions with higher doses could yield more pronounced effects. It is important to acknowledge that neurobiological changes induced by vitamin D may take time to manifest, akin to the delayed onset of action seen with standard antidepressant medications [132].

However, the generalizability of these findings to different age groups is somewhat limited. The study primarily focused on young to middle-aged adults, leaving a gap in our understanding of the potential benefits of vitamin D supplementation for older adults, a demographic with a higher prevalence of depression [132].

Additionally, the co-supplementation of calcium in some trials introduces questions about its impact on the neurophysiological effects of vitamin D in the central nervous system. This aspect warrants further exploration to disentangle the potential synergistic or antagonistic effects of these two nutrients [132].

One intriguing finding from this research is the potential of vitamin D supplementation to reduce perinatal depressive symptoms, underscoring its significance for maternal and fetal health. However, it’s important to note that more research is needed in this specific area to draw precise conclusions and inform clinical practice effectively [132].

In summary, while the study offers promising insights into the role of vitamin D in alleviating depressive symptoms, it also highlights the complexities and nuances involved in its therapeutic use. These findings underscore the need for further research to better understand the optimal conditions for vitamin D supplementation and its potential benefits across diverse demographic groups.

In addition to supplementing vitamin D, psychosocial interventions should be implemented for the improvement of physical activity, an adequate diet, quitting bad habits such as smoking, and maximizing sun exposure for the prevention and treatment of vitamin D deficiency [25].

### Limitations

Possibly, one of the limitations of this study is the small sample size reviewed using only one database. Another limitation is the nature of the study, as compared to a prospective study, a literature review can only review existing studies as opposed to concluding original research. It should be noted that the studies included in the present review were conducted in different populations and used different dosages and intervals for supplementation; thus, the results may have been incomparable, which is a limitation of the current analysis.

## 5. Conclusions

Vitamin D, a fat-soluble vitamin, has emerged as a significant factor in mental health, demonstrating a notable influence on the incidence and prognosis of depression. Several studies have indicated a favorable association between vitamin D levels and a reduced risk of depression. Additionally, there is evidence to suggest that maintaining optimal vitamin D status may contribute to better outcomes for individuals already grappling with depression. This dual role of vitamin D in mitigating both the onset and progression of depression underscores its potential significance in psychiatric healthcare.

However, while vitamin D supplementation has shown promise in alleviating depressive symptoms, several factors remain to be explored to optimize its therapeutic efficacy. Key variables include determining the appropriate dosage and duration of vitamin D supplementation to achieve the desired mental health outcomes. Variations in age groups may also influence the response to vitamin D treatment, necessitating tailored approaches for different demographic groups. Moreover, the impact of co-supplementation with other nutrients or compounds requires careful investigation to assess potential synergistic or antagonistic effects.

The intricate interplay between vitamin D and neuropsychiatric conditions, while promising, highlights the need for further research. It is imperative to determine the precise mechanisms through which vitamin D influences mental health and to identify the specific subpopulations that stand to benefit the most from vitamin D supplementation. This ongoing scientific inquiry is pivotal for the therapeutic potential of vitamin D, ultimately offering new ways for the prevention and management of depression and related neuropsychiatric disorders. As our understanding of the vitamin D and mental health association grows stronger, it holds the promise of improving the well-being and quality of life for individuals confronting these pervasive and often debilitating conditions.

In conclusion, our results suggest that vitamin D deficiency clearly has implications for a vast number of diseases, including mental illness. Compared to the general population, psychiatric patients had a higher frequency of low vitamin D. The most relevant results showing the damage done by vitamin D deficiency were in studies with psychotic spectrum diseases, depression, neurodevelopmental, and neurocognitive diseases. Taken together, we found modest evidence that vitamin D is an effective treatment for symptom reduction or that vitamin supplementation should be considered as a prophylactic treatment.

## Figures and Tables

**Table 1:** Summarization of vitamin D effects in psychiatric diseases.

Psychiatric Diagnosis	Mechanism and Receptors
Psychotic spectrum diseases	Vitamin D has an important role in cell differentiation, neurotransmitter synthesis, calcium homeostasis, cognitive function, prevention of oxidative damage, and neuron function. Receptor—1,25(OH)2D3 antagonizing the effects of the glucocorticoid on hippocampal neurons [59]
	Lower level of serum Vitamin D is caused by long periods of institutionalization, inactivity, and, therefore, less exposure to sunlight [49]
Bipolar disorders	Increased 25OHD synthesis during decompensation [54] Lower level of Vitamin D in depressive patients [55]
Depression	Mechanisms implied are exerting influence on neuroendocrine, immunological, and neurotrophic systems [59]
	Receptors are localized in the hypothalamus [124]
Anxiety disorders	Imbalanced calcium homeostasis [125]
	Interference in serotonin synthesis by expression of the serotonin-synthesizing gene, therefore maintaining serotonin within the normal range [126]
	Mediation of several pathways for insulin or serotonin which were associated with mood disorders [127]
Obsessive-compulsive disorder	Elusive underlying mechanism [80]
Trauma- and stress-related disorders	Vitamin D regulates neuro-inflammatory and neuro-immunological mechanisms [82]
	Brain areas with altered activity in patients with PTSD express vitamin D receptors in the prefrontal cortex, cingulate cortex, and hypothalamus [9]
	Vitamin D mediates the regulation of serotonin and catecholamine [83]
Eating disorder	Vitamin D deficiency as a consequence [87]
Sleep-wake disorder	Vitamin D deficiency is associated with lower sleep duration, worse sleep quality, an increase in time of sleep onset in pediatric populations, and circadian rhythm regulation [95]
Elimination disorders	Prostatic cells can express a hydroxylase that can synthesize the active form of vitamin D [90]
	Vitamin D receptors are located in the urinary bladder and pelvic floor muscles [91]
Sexual disorders	Low levels of vitamin D are associated with an increased risk for atherosclerotic events through inflammation, endothelial dysfunction, atherosclerosis, and impaired glucose metabolism [104]
	Receptors of 1,25(OH)2-D3 inhibit the expression of inducible nitric oxide NO synthetase [128]
Neurocognitive disease	Important role in memory formation; the active form of vitamin D supports neurotransmission, neuroprotection, and synaptic plasticity [110]
	Vitamin D receptors are present in the hippocampus. They are responsible for blocking calcium influx and also the toxicity in cultured mesencephalic neurons or hippocampal neurons [129]

## Data Availability

Not applicable.
